# First report of the complete mitochondrial genome of an *Ophidascaris* species from the European hedgehog (*Erinaceus europaeus*) in China

**DOI:** 10.1016/j.ijppaw.2025.101177

**Published:** 2025-12-15

**Authors:** Wei Hu, Ying Xun, Rong Cheng, Tian-Yin Cheng, Lei Liu, Guo-Hua Liu

**Affiliations:** Research Center for Parasites & Vectors, College of Veterinary Medicine, Hunan Agricultural University, Changsha, Hunan Province, 410128, China

**Keywords:** *Ophidascaris*, Mitochondrial genome, Mitochondrial DNA, Phylogenetics, Ascaridoidea

## Abstract

Species of the genus *Ophidascaris* are zoonotic nematodes primarily parasitic in snakes, but limited genomic resources have hindered phylogenetic resolution and species delineation. To date, no ascarid nematodes have been documented in hedgehogs, making this finding noteworthy. In this study, the complete mitochondrial genome of *Ophidascaris* sp. larvae recovered from European hedgehogs (*Erinaceus europaeus*) in China was sequenced using Illumina technology, annotated, and compared with published sequences. The mitogenome (14,624 bp) contains 12 protein-coding genes, 22 tRNAs, two rRNAs, and—as in other nematodes—lacks the atp8 gene. Comparative analyses showed nucleotide divergence (14.4 %–17.1 %) from *O. wangi* and *O. baylisi*, supported its distinct genetic identity. Phylogenetic analyses confirmed its placement within the *Ophidascaris* genus with strong statistical support. This study provides the first complete mitogenome of an *Ophidascaris* species recovered from a hedgehog and suggests that hedgehogs may serve as intermediate hosts, thereby expanding the known host range. The mitogenome generated here provides valuable molecular markers for species identification, phylogenetic reconstruction, and future epidemiological surveillance.

## Highlights

• First complete mitogenome of an *Ophidascaris* species from the hedgehog.
• Hedgehogs identified as a potential intermediate host for *Ophidascaris*.
• Mitogenome reveals 14–17 % divergence from known *Ophidascaris* species.
• Phylogenomics supports a distinct lineage within the genus *Ophidascaris*.
• New genomic data enhance molecular diagnosis and zoonotic surveillance.

## Graphical abstract

**Figure:**
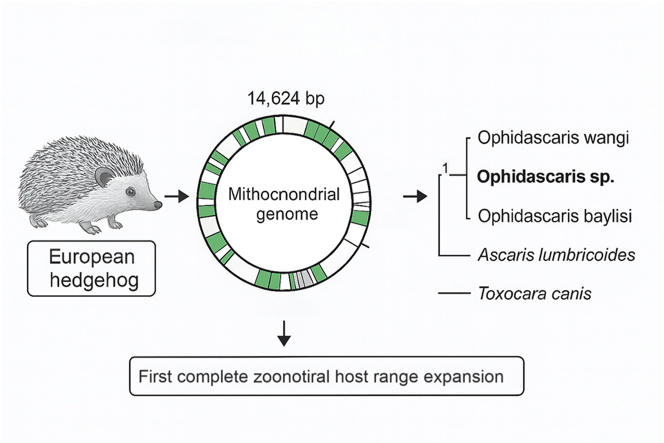

## 1 Introduction

The genus *Ophidascaris* (Ascaridida: Ascarididae) comprises parasitic nematodes with a cosmopolitan distribution, primarily utilizing snakes as their definitive hosts (Elbihari and Hussein, 1973). Beyond their veterinary impact in reptiles, these parasites are of emerging zoonotic concern. Although human infections are rare, they can lead to severe neural larva migrans or eosinophilic meningoencephalitis following accidental ingestion of infective larvae (Hossain et al., 2023). The complex life cycle of *Ophidascaris* typically involves small mammals as intermediate hosts, within which larvae encyst in tissues before being transmitted to the definitive snake host (Sprent, 1970). This ecological trait highlights the possibility of unexpected host associations and emphasizes the need for continued surveillance.

Despite their medical and ecological significance, the molecular characterization of *Ophidascaris* species remains limited. To date, complete mitochondrial genome (mitogenome) datasets are available for only two of the more than 30 described species: *O. wangi* and *O. baylisi* (Li et al., 2016). Such datasets are essential for phylogenetic inference and species delimitation, yet the scarcity of complete mitogenomes has limited progress in resolving evolutionary relationships within the genus.

The European hedgehog (*Erinaceus europaeus*) is a well-studied host for various helminths, including *Crenosoma striatum* and *Capillaria* spp. (Riley and Chomel, 2005). However, no members of the family Ascarididae have previously been documented in hedgehogs, making the detection of *Ophidascaris* larvae particularly noteworthy.

In this study, we recovered larval nematodes from European hedgehogs in China that were morphologically consistent with the genus *Ophidascaris*. This provided a unique opportunity to investigate a potential novel host-parasite association. To confirm its taxonomic placement and assess the phylogenetic relationships of this isolate, we sequenced and characterized its complete mitogenome. Our objectives were to: (1) provide the first mitogenome of an *Ophidascaris* species from a hedgehog; (2) compare its genetic features and divergence with congeneric species; and (3) elucidate its phylogenetic position within the Ascaridoidea. This work provides essential genomic resources that will facilitate species identification, epidemiological studies, and a deeper understanding of the evolution and host range of this enigmatic parasite genus.

## 2 Materials and methods

### 2.1 Sample collection and necropsy

Between June 2021 and October 2024, 71 moribund European hedgehogs (*Erinaceus europaeus*) were collected from Huarong, Hunan, Xixian and Jiaxian, Henan, China. Necropsies were performed immediately upon admission to ensure minimal tissue degradation. The abdominal cavity and major organs were systematically examined, and the stomach and intestines were flushed with 0.7 % saline. Sediments were inspected for helminths, and the number and location of nematode larvae were recorded for each animal.

To address potential co-infections, all larvae were inspected morphologically under stereomicroscopy, and only the encysted nematodes found in hepatic or mesenteric tissues were included in subsequent analyses.

### 2.2 Sample collection and DNA extraction

Larval nematodes were collected from naturally infected European hedgehogs examined during this survey. Specimens were washed in physiological saline, fixed in 70 % ethanol, and stored at −20 °C until DNA extraction. As L3 larvae lack sufficient morphological characteristics for reliable identification, molecular analyses were conducted using the nuclear 18S rRNA gene and the mitochondrial *cox1* region.

Genomic DNA was extracted from individual larvae using SDS/proteinase K lysis followed by spin-column purification (Wizard® SV Genomic DNA Purification System, Promega). To assess possible mitochondrial variation among larvae, partial *cox1* fragments were amplified and sequenced from several individuals. All obtained sequences were identical, indicating that the larvae belonged to a single mitochondrial lineage.

### 2.3 Amplification and sequencing of *cox1* and 18S rRNA gene

Partial *cox1* and 18S rRNA gene fragments were amplified using primer pairs JB3/JB4.5 (Zhu et al., 2000) and Nem_18S_F/Nem_18S_R (Floyd et al., 2005), respectively. All primers were synthesized and purified at OPC grade (Sangon Biotech, Shanghai, China).

PCR reactions were performed in a 25 μL total volume containing 1 μL genomic DNA, 12.5 μL 2 × Master Mix, 1 μL of each primer (10 μM stock; final concentration 0.4 μM), and nuclease-free water. Thermal cycling was carried out as follows: 95 °C for 6 min; 35 cycles of 95 °C for 30 s, 53 °C for 30 s, and 72 °C for 1 min; followed by a final extension at 72 °C for 6 min.

Target bands were excised and purified using the EasyPure Quick Gel Extraction Kit (TransGen, China). DNA fragments were cloned using the pGEM-T Easy Vector System I (Promega, USA) following the manufacturer's instructions. The ligation reaction (10 μL total) contained 1 μL pGEM-T vector (50 ng/μL), 3 μL purified PCR product, 1 μL 10 × T4 DNA ligation buffer, 1 μL T4 DNA ligase (3 U/μL), and 4 μL ddH_2_O. The mixture was gently mixed and incubated at 16 °C for 6 h. The ligation products were transformed into *E. coli* DH5α competent cells (purchased from Beijing All-Gold Biotechnology Co., China) using standard heat-shock transformation and cultured overnight at 37 °C on LB agar plates containing 100 μg/mL ampicillin, 40 μg/mL X-Gal, and 0.5 mM IPTG for blue–white screening.

Positive white colonies were selected and inoculated into LB broth for overnight culture. Recombinant plasmids were extracted using the EasyPure Plasmid MiniPrep Kit (TransGen Biotech, China) according to the manufacturer's instructions. Insert verification was performed by restriction enzyme digestion and Sanger sequencing (Tsingke Biotechnology, Beijing, China).

The resulting sequences were assembled using DNAMAN v6.0 (Lynnon BioSoft, USA). The partial 18S rRNA gene and *cox1* sequences of *Ophidascaris* sp. were deposited in the National Center for Biotechnology Information (NCBI: https://www.ncbi.nlm.nih.gov/) GenBank database under accession numbers PV687032.1 and PX116874, respectively. Homology searches were subsequently performed using the Basic Local Alignment Search Tool (BLAST: https://blast.ncbi.nlm.nih.gov/Blast.cgi) against the GenBank database to confirm the taxonomic status of the specimens.

### 2.4 Mitochondrial genome sequencing and assembly

Genomic DNA for mitochondrial genome sequencing was extracted from a pooled sample of ten L3 larvae. Pooling was carried out only after cox1 sequencing showed that all individuals shared an identical haplotype, ensuring that no mixed mitochondrial lineages were present. The ten larvae used for sequencing were collected from three heavily infected hedgehogs in Huarong, Hunan Province, in July 2024.

DNA concentration and purity were assessed spectrophotometrically. Sequencing libraries were prepared using the NEBNext® Ultra™ II DNA Library Prep Kit (New England Biolabs, USA) with an average insert size of ∼350 bp. Paired-end 150 bp reads (PE150) were generated on an Illumina NovaSeq 6000 platform (Majorbio Bio-Pharm Technology Co., Ltd., Shanghai, China).

Raw reads were processed with Fastp v0.19.7 to remove adapters, low-quality bases, and ambiguous reads. The mitochondrial genome was assembled in Geneious Prime v2023.2.1 using the cox1 gene as a seed reference, with a minimum overlap of 180 bp and 100 % identity. Circularization was confirmed by identifying overlapping terminal regions. Gene annotation was carried out using ORFfinder, tRNAscan-SE, ARWEN, and the MITOS web server, and the circular genome map was generated with Proksee. The primers used for validating the mitochondrial genome assembly are summarized in Table 1.

**Table 1:** Validation primers designed for the assembly of the mitochondrial genome of *Ophidascaris* sp.

Primer	Sequence(5′-3′)	Genes in amplified region	Expected size (kb)
OF1-F	ACTTGGATTCATTGAGTGGGC	*rrnS* ∼ trnS2^UCÑ^ LNCR ∼ trnÑ trnY ∼ *nad1*	2.7
OF1-R	AGCCCCTAACCAACTCACTTT		
OF2-F	GGTGAAAGTGAGTTGGTTAGG	*nad1*∼*atp6*∼trnK ∼ trnL2^UUR^ ∼ trnS2^AGÑ^ *nad2*∼trnI ∼ trnR ∼ trnQ ∼ trnF ∼ *cytb*	2.5
OF2-R	CAATAGAAGTCCCTCACACAG		
OF3-F	TTAGGTCCTGTAGATGGCGGT	trnL1^CUÑ^ *cox*3∼trnT ∼ *nad*4	2.0
OF3-R	CACAGGAACCGAAAACCCAGT		
OF4-F	AGAGTGATGCTAAGGCGTTGG	*nad4*∼SNCR ∼ *cox1*∼trnC ∼ trnM ∼ trnD ∼ trnG ∼ *cox2*∼trnH ∼ *rrnL*	3.6
OF4-R	GTCCTCACGCTAAGACTGCCA		
OF5-F	TAGTATGAATGGGGTTTTGGCT	*rrn*L *∼ nad3*∼*nad5*	1.7
OF5-R	ATTACGACCATCCTGCTGACC		
OF6-F	GGGTCAGCAGGATGGTCGTAA	*nad5*∼trnÃ trnP ∼ trnV ∼ *nad6*∼*nad4L* ∼ trnW ∼ trnE ∼ *rrnS*	2.1
OF6-R	TACTGTCTTTTACATTTTCCGCCC		

### 2.5 Mitochondrial genome annotation

The assembled mitogenome of *Ophidascaris* sp. was aligned with the published genome of *O. wangi* (GenBank accession no. MK106624.1) using MAFFT v7.123 (Kazutaka and Daron M, 2013) to delineate gene boundaries. The nucleotide sequences of the 12 protein-coding genes (PCGs) were translated using the invertebrate mitochondrial genetic code in MEGA v7 (Kumar et al., 2016), and start/stop codons were verified by comparison with *O. wangi*. Transfer RNA (tRNA) genes were identified using tRNAscan-SE (Chan et al., 2021), and ribosomal RNA (rRNA) genes were annotated with the MITOS WebServer (Bernt et al., 2013). The complete annotated mitogenome has been deposited in GenBank under accession number PP133902.1.

**Figure_1:**
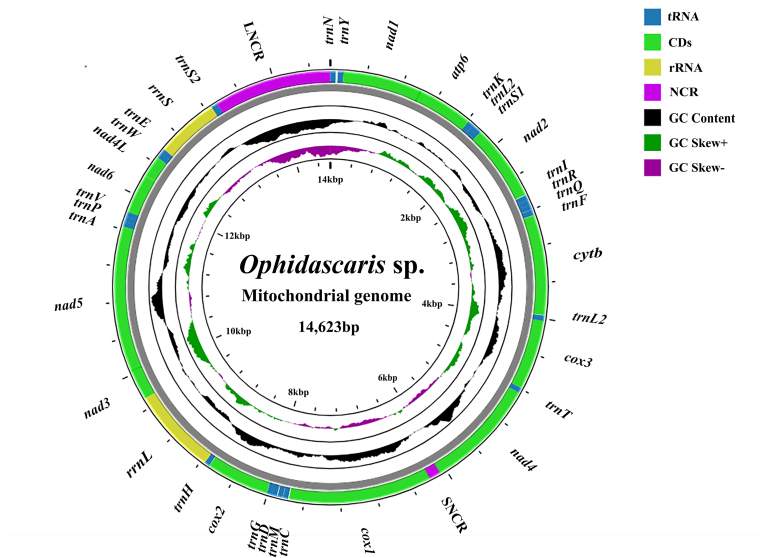
Circular mitochondrial genome map of *Ophidascaris* sp. (14,623 bp), comprising 12 protein-coding genes, 22 tRNA genes, two rRNA genes, and two non-coding regions. Genes are arranged clockwise (5′→3′). Colors indicate functional categories. The inner rings display GC content (black) and GC skew (green/purple). Both metrics were calculated using a sliding window (window size 300 bp, step size 30 bp). GC skew was computed as (G − C)/(G + C), with positive values shown in green and negative values in purple.

The circular mitochondrial genome map (Fig. 1) was generated using Proksee. Because GC content and GC skew were calculated using a sliding-window approach, the profiles reflect local nucleotide composition rather than gene-level averages. As a result, short segments of the genome may display negative GC skew even when the corresponding gene-level values in Table 3 are positive. This also explains why GC content appears as a continuous landscape across the genome, rather than as deviations from a single mean value.

### 2.6 Comparative analyses of nucleotide and amino-acid divergences among mitochondrial genes

Pairwise divergences at the nucleotide (nt) and amino-acid (aa) levels were calculated for each mitochondrial gene among *Ophidascaris* sp. (OS), *O. baylisi* (OB), and *O. wangi* (OW). For nt-level comparisons, each gene was aligned using MAFFT v7 with default parameters, and uncorrected p-distances (percentages) were calculated in MEGA v7. For aa-level comparisons, the 12 PCGs were translated using the invertebrate mitochondrial code, aligned separately, and analyzed for p-distance percentages. Ribosomal genes (*rrnL*, *rrnS*) were analyzed only at the nucleotide level.

### 2.7 Phylogenetic analysis

Concatenated amino acid sequences of the 12 protein-coding genes (PCGs) from the mitogenome of *Ophidascaris* sp. were aligned with those of previously reported species. The dataset comprised representatives of Anisakidae: *Pseudoterranova bulbosa* (KU558720), *P. cattani* (KU558721), *P. krabbei* (KU558724), *P. azarasi* (NC027163), *Anisakis pegreffii* (LC222461), *A. simplex* (NC007934), *Contracaecum rudolphii* (NC014870), *C. ogmorhini* (KU558725), *C. osculatum* (NC024037), and *Ortleppascaris sinensis* (KU950438); Ascarididae: *Ascaris* sp. (KC839987), *A. ovis* (KU522453), *A. suum* (HQ704901), *Parascaris univalens* (NC024884), *P. equorum* (MF678786), *Baylisascaris transfuga* (NC015924), *B. schroederi* (NC015927), *B. procyonis* (NC016200), *Ophidascaris wangi* (MK106624), *O. baylisi* (MW880927), and *Toxascaris leonina* (NC023504); as well as *Toxocara cati* (AM411622), *T. malaysiensis* (AM412316), and *T. canis* (AM411108); Heterakidae: *Heterakis beramporia* (KU529972) and *H. gallinarum* (KU529973); and Ascaridiidae: *Ascaridia* sp. (JX624730) and *A. columbae* (NC021643). *Enterobius vermicularis* (Oxyuridae; NC011300) was used as the outgroup.

Gene-wise amino acid alignments were generated in MAFFT v7.123 and concatenated. Ambiguously aligned regions were excluded with Gblocks v0.91b (http://phylogeny.lirmm.fr/) using default parameters with relaxed flanking positions. The best-fitting substitution model (JTT + F + I + G) was determined with ModelFinder in IQ-TREE under the Akaike information criterion (AIC).

Maximum likelihood (ML) analyses were conducted with PhyML v3.0(Guindon et al., 2009) and IQ-TREE (Nguyen et al., 2015) under the selected model, with nodal support estimated from 2000 ultrafast bootstrap replicates. Bayesian inference (BI) was performed in MrBayes v3.1.1 (Ronquist et al., 2012) with two independent runs of four chains each for 2,000,000 generations, sampling every 1000 generations. The first 25 % of sampled trees were discarded as burn-in. Convergence was assessed by a potential scale reduction factor (PSRF ≈ 1.0) and an average standard deviation of split frequencies <0.01. Phylogenetic trees were visualized using FigTree v1.4.2 (Chen et al., 2014).

## 3 Results

### 3.1 Sampling and parasite recovery

Among the 71 hedgehogs examined, 45 (63.4 %) harbored encysted nematode larvae. Prevalence differed among location, being highest in Huarong, Hunan (17/19, 89.5 %), intermediate in Xixian, Henan (28/43, 65.1 %), and absent in Jiaxian, Henan (0/9). Infection intensity ranged from 1 to 74 larvae per animal. Larvae were consistently located on the liver surface, within the liver parenchyma, and along the mesentery, and all were enclosed within well-defined fibrous cysts. No free larvae were observed.

### 3.2 Molecular identification of *Ophidascaris* sp

The partial 18S rRNA gene sequence obtained from the larval nematodes showed >90 % identity with reference sequences of *Ascaris lumbricoides* (GenBank accession U94366.1), confirming that the specimens belong to the superfamily Ascaridoidea. However, due to the high conservation of the 18S rRNA gene among ascaridoid nematodes, this marker is typically used for identification at higher taxonomic levels, such as superfamily (Liu et al., 2016), and is not suitable for distinguishing species within the genus *Ophidascaris*.

In contrast, the mitochondrial cytochrome *c* oxidase subunit 1 (*cox1*) gene, which exhibits greater variation, was used for species-level identification (Palomares-Rius et al., 2017). The partial *cox1* sequence of the present specimens exhibited 10–12 % nucleotide divergence compared to *O. wangi* and *O. baylisi*, with an overall similarity of approximately 88–90 %. Previous studies have suggested that such mitochondrial sequence differences typically reflect interspecific variation within the Ascarididae family (Zhao et al., 2022), indicating that the genetic divergence observed here is sufficient to distinguish these species.

Taken together, the 18S rRNA sequence confirmed placement within Ascaridoidea, whereas the mitochondrial *cox1* gene provided species-level resolution, indicating that the isolates belong to *Ophidascaris* but do not match any described species. Considering the absence of adult specimens and the limited availability of reference sequences for *Ophidascaris* in GenBank, the isolates are provisionally designated as *Ophidascaris* sp., pending confirmation with additional morphological and molecular evidence.

### 3.3 General mitochondrial genome features

The complete mitogenome of *Ophidascaris* sp. (GenBank accession no. PP133902.1) is a circular DNA molecule of 14,624 bp (Fig. 1), comprising 36 genes: 12 PCGs (*atp6*, *cytb*, *cox1–3*, *nad1–6*, and *nad4L*), 22 tRNA genes, two rRNA genes, and two non-coding regions (NCRs). As is typical for nematode mitogenomes, the atp8 gene is absent (Table 2). All genes are encoded on the same strand, with the canonical GA3 gene arrangement (Jabbar et al., 2014).

**Table 2:** Organization of the mitochondrial genome of *Ophidascaris* sp.

Gene	Positions(bp)	Size(bp)	Amino acids (aa)	Start/Stop codon	Anticodon	Intergenic region(bp)
tRNA-Asn(N)	1–58	58			GTT	0
tRNA-Tyr(Y)	82–142	61			GTA	+23
*nad1*	143–1015	873	290	TTG/TAG		0
*atp6*	1020–1619	600	199	ATT/TAG		+4
tRNA-Lys(K)	1619–1689	71			TTT	−1
tRNA-Leu^UUR^(L_2_)	1682–1740	59			TAA	−8
tRNA-Ser^AGN^(S_1_)	1737–1790	54			TCT	−4
*nad2*	1791–2634	664	221	GTG/T		0
tRNA-Ile(I)	2634–2690	57			GAT	−1
tRNA-Arg(R)	2690–2745	56			ACG	−1
tRNA-Gln(Q)	2745–2799	55			TTG	−1
tRNA-Phe(F)	2803–2862	60			GAA	+3
*cytb*	2862–3963	1102	367	ATT/T		−1
tRNA-Leu^CUN^(L_1_)	3966–4021	56			TAG	+2
*cox3*	4022–4789	768	255	TTG/TAG		0
tRNA-Thr(T)	4790–4843	54			TGT	0
*nad4*	4845–6074	1230	409	TTG/TAA		+1
SNCR	6075–6194	120				0
*cox1*	6195–7770	1576	525	TTG/T		0
tRNA-Cys(C)	7771–7825	55			GCA	0
tRNA-Met(M)	7830–7889	60			CAT	+4
tRNA-Asp(D)	7898–7952	55			GTC	+8
tRNA-Gly(G)	7953–8030	78			TCC	0
*cox2*	8008–8701	694	231	ATT/T		−23
tRNA-His(H)	8702–8757	56			GTG	0
*rrnL*	8758–9709	952				0
*nad3*	9713–10048	336	111	TTG/TAG		+3
*nad5*	10051–11632	1582	527	ATT/T		+2
tRNA-Ala(A)	11632–11687	56			TGC	−1
tRNA-Pro(P)	11688–11744	57			TGG	0
tRNA-Val(V)	11744–11797	54			TAC	−1
*nad6*	11798–12232	435	143	TTG/TAG		0
*nad4L*	12235–12468	234	76	ATG/TAG		+2
tRNA-Trp(W)	12470–12525	56			TCA	+1
tRNA-Glu(E)	12524–12580	57			TTC	−2
*rrnS*	12581–13267	687				0
tRNA-Ser^UCN^(S_2_)	13268–13337	70			TGA	0
LNCR	13338–14624	1287				0

Notes: Incomplete stop codons are indicated as T and are presumed to be completed by post-transcriptional polyadenylation. Positive intergenic values indicate the number of nucleotides between adjacent genes, whereas negative values indicate overlapping nucleotides.

The overall A + T content is 70.19 % (T: 48.63 %, A: 21.57 %, G: 21.38 %, C: 8.43 %), slightly higher than that of *O. wangi* (69.24 %; MK106624) and O. baylisi (69.98 %; MW880927). Whole-genome AT and GC skew values are −0.39 and 0.43, respectively (Table 3). Gene order and transcriptional orientation are identical to those of *O. wangi* and *O. baylisi*. In addition to these genome-wide features, the GC content and GC skew profiles shown in Fig. 1 were generated using a sliding-window analysis (300-bp window, 30-bp step). Because the sliding-window approach captures local rather than gene-level nucleotide composition, short genomic regions may display negative GC skew even though all gene-level values are positive (Table 3).

**Table 3:** Nucleotide composition and skews of the *Ophidascaris* sp. mitochondrial genome.

Gene	Nucleotide frequencey				A + T(%)	AT-skew	GC-skew
	A(%) G(%) T(%) C(%)						
*atp6*	19.00	22.33	51.67	7.00	70.67	−0.46	0.52
*cox1*	18.46	23.10	47.53	10.91	65.99	−0.44	0.37
*cox2*	19.60	25.07	46.69	8.65	66.28	−0.41	0.49
*cox3*	16.67	24.48	50.78	8.07	67.45	−0.51	0.50
*cytb*	16.52	24.32	50.54	8.62	67.06	−0.51	0.48
*nad1*	18.67	21.65	49.71	9.97	68.38	−0.45	0.37
*nad2*	16.82	22.99	54.27	5.92	71.09	−0.53	0.59
*nad3*	18.15	25.60	52.38	3.87	70.54	−0.49	0.74
*nad4*	16.75	22.03	51.46	9.76	68.21	−0.51	0.37
*nad4L*	20.09	23.08	52.99	3.85	73.08	−0.45	0.71
*nad5*	17.57	21.55	53.10	7.77	70.67	−0.50	0.47
*nad6*	17.24	17.47	58.16	7.13	75.40	−0.54	0.42
*rrnS*	29.11	21.11	39.74	10.04	68.85	−0.15	0.36
*rrnL*	23.11	19.54	50.00	7.35	73.11	−0.37	0.45
22 tRNAs	28.49	21.24	41.39	8.88	69.88	−0.18	0.41
SNCR	34.17	20.83	39.17	5.83	73.33	−0.06	0.56
LNCR	39.08	11.81	40.07	8.39	79.80	−0.02	0.17
Total	21.57	21.38	48.63	8.43	70.19	−0.39	0.43

Notes: AT-skew = (A – T)/(A + T); GC-skew = (G – C)/(G + C). Gene names are abbreviated as follows: PCGs (*atp*6, *cox*1–3, *cytb*, *nad*1–6, *nad4L)*, ribosomal RNAs (*rrn*S, *rrn*L), transfer RNAs (22 tRNAs), and non-coding regions (SNCR, LNCR).

### 3.4 Protein-coding genes and codon usage

The 12 PCGs of the *Ophidascaris* sp. mitogenome encode 3416 amino acids. Four initiation codons were identified (TTG, GTG, ATT, and ATG). TTG was the most frequent, initiating seven genes (*cox1*, *cox2*, *cox3*, *nad1*, *nad3*, *nad4*, and *nad6*). ATT initiated *nad5* and *atp6*, ATG initiated *cytb* and *nad4L*, and GTG initiated *nad2*.

Three termination codons were detected (TAG, TAA, and incomplete T). The incomplete T occurred in *cox1*, *cox2*, *nad2*, *nad5*, *atp6*, and *cytb*. TAG terminated *cox3*, *nad1*, *nad3*, *nad4L*, and *nad6*; *nad4* ended with TAA. Nucleotide composition and skew values for each PCG are summarized in Table 3.

### 3.5 tRNA and rRNA genes

A total of 22 tRNA genes were identified, ranging from 53 to 78 bp in length (Table 2). Predicted secondary structures fell into three categories. tRNA-Ser^UCN^ exhibited the typical cloverleaf structure; tRNA-Ser^AGN^ and tRNA-Pro lacked the DHU arm but retained the TΨC arm, corresponding to a D-arm–reduced structure; and the remaining 19 tRNAs lacked the TΨC arm, which was replaced by a TV-replacement loop.

Two rRNA genes were also detected: *rrnL* (952 bp), located between tRNA-His and *nad3*, and *rrnS* (687 bp), located between tRNA-Glu and tRNA-Ser^UCN^. The A + T contents of *rrnL* and *rrnS* were 73.11 % and 68.85 %, respectively.

### 3.6 Non-coding regions

The mitogenome contains two major NCRs: a long NCR (LNCR, 1287 bp) located between tRNA-Ser^UCN^ and tRNA-Asn, and a short NCR (SNCR, 120 bp) between *nad4* and *cox1* (Fig. 1, Table 2). The A + T contents of the LNCR and SNCR were 79.80 % and 73.33 %, respectively (Table 3).

### 3.7 Comparative analyses of *Ophidascaris* sp., *O.baylisi* and *O.wangi* mitogenomes

Comparative analyses of the mitogenomes of *Ophidascaris* sp. (OS), *O. baylisi* (OB), and *O. wangi* (OW) showed highly similar gene content and arrangement, with total lengths ranging from 14,624 to 14,784 bp (Table 4). Overall nucleotide divergence was substantial, ranging from 14.43 % between OS and OW to 17.12 % between OS and OB, whereas amino-acid divergence across the 12 PCGs was much lower (5.44–7.72 %), indicating strong functional constraints at the protein level.

**Table 4:** Comparison of nucleotide (nt) and/or predicted amino acid (aa) sequence between mitochondrial genomes of *Ophidascaris* sp. (OS), *Ophidascaris baylisi* (OB), *Ophidascaris wangi* (OW).

Gene/region	Nt length (bp)			Nt diversity (%)			Amino acid no.			Amino acid diversity (%)		
	OS	OB	OW	OS/OB	OS/OW	OB/OW	OS	OB	OW	OS/OB	OS/OW	OB/OW
*cox1*	1576	1578	1578	11.87	9.90	10.71	525	525	525	3.43	1.90	3.81
*cox2*	667	696	696	9.51	8.65	11.35	222	231	231	3.90	1.73	5.19
*nad3*	339	336	333	10.12	10.81	12.61	112	111	110	6.31	6.31	7.27
*nad5*	1573	1582	1582	13.97	11.13	14.66	524	527	527	8.35	6.64	7.59
*mad6*	435	435	435	13.56	13.56	14.02	144	144	144	10.42	10.42	11.11
*nad4L*	234	234	234	11.54	11.97	11.54	77	77	77	9.09	6.49	6.49
*nad1*	873	873	873	12.60	12.83	14.09	290	290	290	6.90	6.90	6.21
*atp6*	616	600	600	9.00	9.33	10.33	205	199	199	5.03	6.03	5.53
*nad2*	835	844	844	15.17	11.61	14.93	278	281	281	12.81	6.05	11.39
*cytb*	1066	1105	1105	15.97	12.61	15.57	355	368	268	11.17	6.99	9.78
*cox3*	768	768	768	13.02	10.81	13.80	255	255	255	0.67	3.14	5.110
*nad4*	1230	1230	1230	15.45	11.79	16.42	409	409	409	9.29	5.13	9.78
*rrnL*	952	956	958	17.41	14.32	15.70						
*rrnS*	687	700	704	12.21	10.89	14.10						
overall	14624	14784	14660	17.12	14.43	17.11	3396	3417	3316	7.72	5.44	7.46

nt = nucleotide; aa = amino acid. Divergence values are uncorrected p-distances (%) calculated in MEGA v7. Amino acid divergences are reported for the 12 protein-coding genes only. Ribosomal genes (*rrn*L, *rrn*S) were analyzed only at the nucleotide level.

Among individual genes, *cox1* and *cox2* were the most conserved, with nucleotide divergence below 12 % and amino acid divergence as low as 1.7–3.9 %, supporting their value as reliable barcoding markers. In contrast, *nad2*, *nad4*, *nad5*, and *cytb* displayed the highest variability, with nucleotide differences exceeding 15 % and amino acid differences approaching 10–13 %, suggesting accelerated evolutionary rates. Ribosomal genes (*rrnL* and *rrnS*) were analyzed only at the nucleotide level, where *rrnL* exhibited the greatest divergence (up to 17.41 %).

### 3.8 Phylogenetic analysis

**Figure_2:**
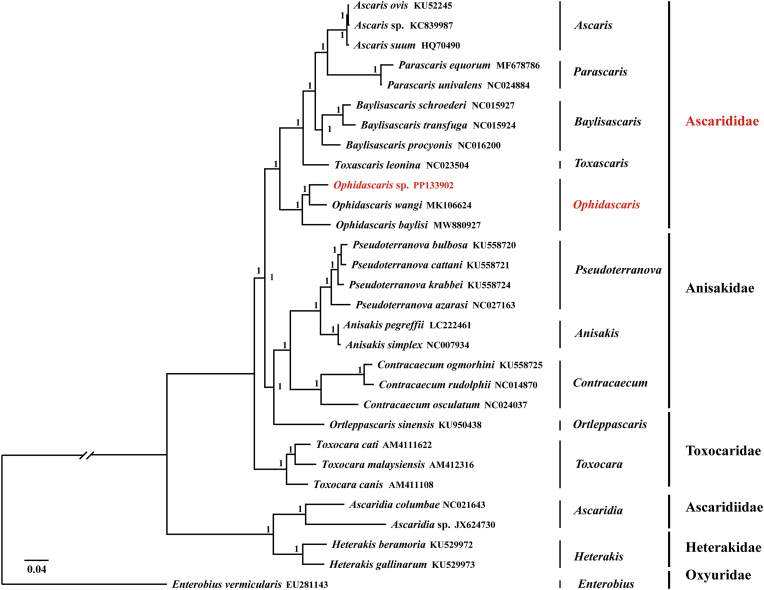
Bayesian inference (BI) phylogenetic tree of *Ophidascaris* sp. and related species (Ascarididae, Anisakidae, Toxocaridae, Heterakidae, and Oxyuridae) based on concatenated amino acid sequences of 12 mitochondrial protein-coding genes. Numbers at nodes indicate Bayesian posterior probabilities; only values ≥ 0.90 are shown. Scale bar represents the number of substitutions per site.Family names are indicated according to current taxonomy.

**Figure_3:**
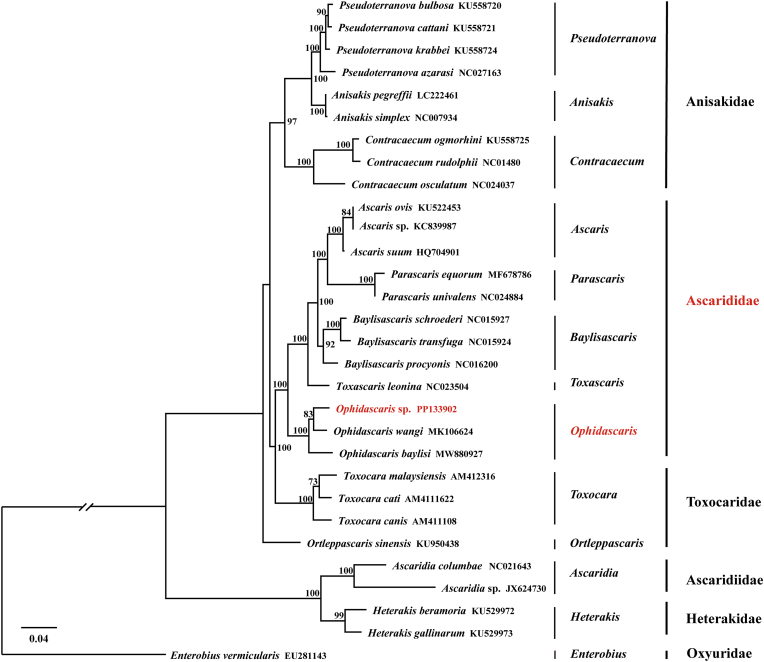
Maximum-likelihood (ML) phylogenetic tree of *Ophidascaris* sp. and related species (Ascarididae, Anisakidae, Toxocaridae, Heterakidae, and Oxyuridae) based on concatenated amino acid sequences of 12 mitochondrial protein-coding genes. Numbers at nodes indicate bootstrap values; only values ≥ 70 % are shown. Scale bar represents the number of substitutions per site. Family names are indicated according to current taxonomy.

Phylogenetic analyses based on the concatenated amino-acid sequences of the 12 mitochondrial protein-coding genes yielded largely congruent BI (Fig. 2) and ML (Fig. 3) topologies, with the major families (Anisakidae, Ascarididae, Toxocaridae, Heterakidae and Ascaridiidae) consistently recovered as well-supported monophyletic groups. In both trees, the newly sequenced *Ophidascaris* sp. clustered with *O. wangi* and *O. baylisi*, forming a strongly supported lineage within Ascarididae.

## 4 Discussion

Our study provides the first complete mitogenome of an *Ophidascaris* species isolated from the European hedgehog, expanding the known host spectrum for this genus. The substantial genetic distance (10–12 %) observed in the *cox1* gene between our isolate and known *Ophidascaris* species strongly suggests that it represents a distinct genetic lineage (Gonçalves et al., 2021). This finding challenges the conventional view of strict host specificity in snake-associated *Ophidascaris* and highlights the potential for cryptic diversity within the genus.

Morphological assessment of the larvae was inherently limited by their early developmental stage and encysted condition. Third-stage *Ophidascaris* larvae lack the key diagnostic characters required for species-level—and even reliable genus-level—identification in adult worms. Critical morphological traits such as lip and interlabial structures, caudal papillae arrangement, and reproductive anatomy are either absent or insufficiently developed at this stage. In addition, the small size and encysted condition of the larvae further obscured fine morphological details. As a consequence, larval morphology alone cannot provide a reliable taxonomic determination within *Ophidascaris*, necessitating the use of molecular markers for generic placement and for evaluating genetic distinctiveness.

The levels of mitochondrial divergence observed here (10–12 % in *cox1* and 14–17 % across the mitogenome) are consistent with interspecific differences within Ascarididae, suggesting that this isolate may represent a previously undescribed lineage of *Ophidascaris*. Nevertheless, because no adult worms were available and species-level diagnosis in this genus traditionally requires adult morphology, we refrain from proposing a new species. Accordingly, we adopt the conservative designation *Ophidascaris* sp., pending the collection of adult specimens and integrated morphological–molecular evidence. This approach avoids premature taxonomic conclusions while acknowledging the possibility of cryptic diversity within the genus.

The most parsimonious explanation for the presence of larval *Ophidascaris* in a hedgehog is that it serves as an intermediate host. This interpretation is supported by the presence of encysted L3 larvae in hepatic and mesenteric tissues, a pattern typical for intermediate hosts rather than paratenic or aberrant hosts. Encystment is consistent with completion of L2→L3 development, as documented in other *Ophidascaris* species (McAllister et al., 1992). Hedgehogs are primarily insectivorous but opportunistically consume small vertebrates, providing a plausible route of infection. No adult worms were recovered; therefore, the hedgehog is not a definitive host. Here, we use “intermediate host” to describe larval development to L3 within the mammalian host.

Recent developments have renewed interest in the zoonotic potential of *Ophidascaris*. In 2023, a landmark case from Australia documented the first human infection with *Ophidascaris robertsi*, in which a live third-stage larva was surgically removed from the brain of an immunocompromised woman (Boehme et al., 2023). This case demonstrates that *Ophidascaris* larvae are capable of aberrant migration in humans and highlights the possibility of cross-species transmission when ecological interfaces between wildlife and humans are disrupted. Although no human infections have yet been reported in China, the trophic pathways required for transmission are present, particularly in regions where snakes are consumed, traded, or kept in captivity, and where wildlife such as hedgehogs forage in peri-urban habitats.

In the Chinese context, hedgehogs may ingest small vertebrates or paratenic hosts carrying *Ophidascaris* larvae, while snakes—especially elapids and colubrids—are widespread across many provinces, creating natural opportunities for completing the parasite's life cycle. Human exposure could theoretically occur through handling wild snakes, consumption of undercooked snake meat, or environmental contamination with nematode eggs shed by infected definitive hosts. From a One Health perspective, the interactions among wildlife (hedgehogs), reptiles (snakes), and humans underscore the need for integrated surveillance. The mitogenomic resources generated in this study provide a molecular foundation for PCR-based detection of *Ophidascaris* in animals and environmental samples, which will be essential for monitoring potential spillover pathways and assessing emerging zoonotic risks in China.

The architecture of the *Ophidascaris* sp. mitogenome is congruent with the conserved pattern observed across Ascaridoidea, including the absence of the *atp8* gene and the characteristic gene order (Gu et al., 2025). To place these findings in a broader evolutionary context, comparison with other ascaridoid mitogenomes shows that this isolate retains the core features shared across the superfamily—such as the GA3 gene order, unidirectional transcription, and the absence of atp8—as seen in *Ascaris*, *Baylisascaris*, *Toxocara*, and *Ascaridia*. Its slightly elevated A + T content suggests subtle lineage-specific compositional shifts (Akshita et al., 2025). Moreover, the higher variability of *nad2*, *nad5*, and *cytb* mirrors trends observed across Ascarididae, supporting their use as informative markers in population-level studies. These shared and unique genomic traits collectively position the new isolate within Ascaridoidea while highlighting features that differentiate it from known *Ophidascaris* species.

Our comparative analysis identified *nad2*, *nad5*, and *cytb* as the most variable genes, making them promising targets for future population genetic and phylogeographic studies to trace the origin and dispersal of this parasite lineage (Jex et al., 2009). The phylogenetic analyses consistently placed *Ophidascaris* sp. in a well-supported clade with *O. wangi* and *O. baylisi*, but the exact position of this clade relative to other Ascarididae varied between the BI and ML trees. In the BI tree, *Ophidascaris* formed a distinct lineage outside the main Ascarididae clade, whereas in the ML tree it was nested within Ascarididae. This pattern, which is broadly consistent with recent mitogenomic studies (Qi et al., 2021; Zhou et al., 2021), indicates that *Ophidascaris* is closely related to ascaridoid nematodes, but its precise placement within or adjacent to Ascarididae remains unresolved. The strong nodal support, even for a larval specimen, underscores the value of mitogenomic data for taxonomic placement when morphological characters are limited or ontogenetically variable.

This finding may have implications for wildlife disease surveillance, particularly in regions where hedgehogs and snakes coexist. In regions like China, where hedgehogs may inhabit peri-urban areas and snakes are consumed or kept as pets, the interface for cross-species transmission is amplified. The genomic resources generated here provide foundational tools for developing PCR-based assays to screen for *Ophidascaris* in potential hosts and environmental samples, enabling proactive surveillance.

Future research should prioritize the collection of adult specimens from sympatric snake populations to morphologically and molecularly match this larval type, thereby closing its life cycle. Furthermore, comparative transcriptomic analyses between larvae from intermediate hosts and adults from definitive hosts could reveal genes involved in host adaptation and pathogenicity. Finally, expanded sampling across the geographical range of *Ophidascaris* will be crucial to unravel its full genetic diversity and test hypotheses about co-evolution with its hosts.

## 5 Conclusion

This study presents the first complete mitogenome of an *Ophidascaris* species from the European hedgehog, demonstrating its distinct genetic identity within the genus and expanding the known host range beyond snakes. The significant nucleotide divergence from *O. wangi* and *O. baylisi*, together with the broader comparative analyses across Ascaridoidea, confirms that this lineage retains the canonical mitogenomic architecture of the superfamily while exhibiting unique genomic signatures that distinguish it from described species. These findings highlight the potential for hedgehogs to serve as intermediate hosts in the life cycle of *Ophidascaris*, with implications for zoonotic transmission in overlapping ecosystems. The mitogenome generated here will facilitate molecular diagnostics, phylogenetic reconstruction, and epidemiological tracking, providing a foundation for integrative taxonomy of this understudied parasite group. Future studies should focus on obtaining adult specimens to validate morphology and explore transmission dynamics.

## CRediT authorship contribution statement

**Wei Hu:** Writing – review & editing, Writing – original draft, Visualization, Resources, Methodology, Investigation, Formal analysis, Data curation, Conceptualization. **Ying Xun:** Writing – review & editing, Resources, Investigation. **Rong Cheng:** Writing – review & editing, Resources, Investigation. **Tian-Yin Cheng:** Supervision, Methodology, Investigation. **Lei Liu:** Supervision, Methodology, Investigation. **Guo-Hua Liu:** Writing – review & editing, Supervision, Project administration, Funding acquisition, Conceptualization.

## Ethics approval and consent to participate

*Ophidascaris* sp. specimens were collected from naturally deceased or moribund hedgehogs submitted for post-mortem examination. According to institutional and national regulations, ethics approval was not required for necropsy of animals that died or were euthanized for clinical reasons unrelated to this study.

## Availability of data and materials

The complete mitochondrial genome sequence of *Ophidascaris* sp. generated in this study has been deposited in GenBank under the accession number PP133902.1. All other data supporting the findings of this study (e.g., alignment files, phylogenetic trees) are available from the corresponding author upon reasonable request.

## Consent for publication

Not applicable.

## Declaration of generative AI and AI-assisted technologies in the writing process

During the preparation of this work, the author(s) used ChatGPT for proofreading and improving the readability of the manuscript. After using this tool, the author(s) reviewed and edited the content as needed and take(s) full responsibility for the content of the published article.

## Source of funding

The study was partially funded by the Science and Technology Innovation Program of Hunan Province (Grant no. 2025RC1053).

## Declaration of competing interest

The authors declare the following financial interests/personal relationships which may be considered as potential competing interests: Guohua Liu reports financial support was provided by Furong Program Furong Program for Leading Talents in Technological Innovation. If there are other authors, they declare that they have no known competing financial interests or personal relationships that could have appeared to influence the work reported in this paper.
